# Cardioprotective effects of polydatin against myocardial injury in diabetic rats via inhibition of NADPH oxidase and NF-κB activities

**DOI:** 10.1186/s12906-020-03177-y

**Published:** 2020-12-11

**Authors:** Ying-Ying Tan, Lei-Xin Chen, Ling Fang, Qi Zhang

**Affiliations:** 1 Shaanxi Key Laboratory of Chinese Medicine Encephalopathy, Shaanxi University of Chinese Medicine, Century Avenue, Xianyang, Shaanxi 712046 P. R. China; 2 Affiliated Hospital of Shaanxi University of Chinese Medicine, Xianyang, Shaanxi 712046 P. R. China

**Keywords:** Polydatin, Diabetic cardiomyopathy, Reactive oxygen species, NADPH oxidase, NF-κB, Diabetes

## Abstract

**Background:**

Diabetic cardiomyopathy is a main cause of the increased morbidity in diabetic patients, no effective treatment is available so far. Polydatin, a resveratrol glucoside isolated from the *Polygonum cuspidatum*, was found by our and others have antioxidant and cardioprotective activities. Therapeutic effects of polydatin on diabetic cardiomyopathy and the possible mechanisms remains unclear. This study aimed to investigate the cardioprotective effects and underlying mechanisms of polydatin on myocardial injury induced by hyperglycemia.

**Methods:**

Diabetes in rats was made by high-fat diet combined with multiple low doses of streptozotocin, and then treated with polydatin (100 mg·kg-1·day-1, by gavage) for 8 weeks. Cardiac function was examined by echocardiography. Myocardial tissue and blood samples were collected for histology, protein and metabolic characteristics analysis. In cultured H9c2 cells with 30 mM of glucose, the direct effects of polydatin on myocyte injury were also observed.

**Results:**

In diabetic rats, polydatin administration significantly improved myocardial dysfunction and attenuated histological abnormalities, as evidenced by elevating left ventricular shortening fraction and ejection fraction, as well as reducing cardiac hypertrophy and interstitial fibrosis. In cultured H9c2 cells, pretreatment of polydatin dose-dependently inhibited high glucose-induced cardiomyocyte injury. Further observation evidenced that polydatin suppressed the increase in the reactive oxygen species levels, NADPH oxidase activity and inflammatory cytokines production induced by hyperglycemia in vivo and in vitro. Polydatin also prevented the increase expression of NOX4, NOX2 and NF-κB in the high glucose -stimulated H9c2 cells and diabetic hearts.

**Conclusions:**

Our results demonstrate that the cardioprotective effect of polydatin against hyperglycemia-induced myocardial injury is mediated by inhibition of NADPH oxidase and NF-κB activity. The findings may provide a novel understanding the mechanisms of the polydatin to be a potential treatment of diabetic cardiomyopathy.

**Supplementary Information:**

The online version contains supplementary material available at 10.1186/s12906-020-03177-y.

## Background

For diabetic patients, cardiovascular complications are the most common cause of morbidity [1]. Diabetic cardiomyopathy manifests initially as cardiac fibrosis, followed by remodeling of dysfunctional myocardium, diastolic dysfunction, systolic dysfunction, and eventually by cardiac failure [2, 3]. The underlying mechanisms have been proposed to result in the pathogenesis of diabetic cardiomyopathy, which include increased oxidative stress, cardiac inflammation, insulin resistance, endoplasmic reticulum stress, etc. [4–9]. The precise mechanisms implicated in diabetic cardiomyopathy remain unclear although the diabetic cardiomyopathy has significant clinical and pathological entity.

It is reported that the oxidative stress results from the reactive oxygen species (ROS) induced by hyperglycemia, has been shown contributing to the injury and inflammation in heart [5, 10]. Accumulating evidences suggest that the primary source of cardiac ROS production comes from nicotinamide adenine dinucleotide phosphate (NADPH) oxidase, and that hyperglycemia directly augments the NADPH oxidases activities in the diabetic myocardium [11–14]. More and more evidences have been shown that diabetic cardiomyopathy is characterized by a significant increase in proinflammatory cytokines in the myocardium [10, 15–17]. It has been reported that the levels of proinflammatory cytokines show a positive correlation with ROS production and decreased ventricular function [15, 17–19]. Thus, targeting oxidative stress- cytokine signaling might provide a new way to treat the diabetic cardiomyopathy.

Recent updates, the therapeutic potential of natural products have been assessed gradually for their therapeutic potential in cardiovascular diseases [20]. Polydatin is a resveratrol glucoside isolated from the perennial herb *Polygonum cuspidatum* as natural compound. A range of diverse biological and pharmacological activities of polydatin in various disease models have been reported, which include antioxidant, anti-inflammatory, and neuroprotective functions [21]. The further studies show that polydatin protects against cardiac ischemia–reperfusion injury by modulating renin-angiotensin system and reducing ROS production [22–24], attenuates the cardiac dysfunction generated by burn through inhibiting ryanodine receptors [25], and alleviates cardiac dysfunction and mitochondrial function in the diabetic model [26]. In line with above findings, our study shows that polydatin protects against angiotensin II-induced cardiac hypertrophy both in vitro and in vivo through suppression of NADPH oxidase activity and superoxide production [27]. In addition, our previous studies have also shown that polydatin inhibits insulin resistance and improves hepatic steatosis [28]. Interestingly, in diabetic models induced by streptozotocin (STZ) injection alone, polydatin is also reported to exert cardioprotective effects through activating PPARβ and inhibiting expressions of NF-κB, COX-2 and iNOS [29], improving mitochondrial bioenergetics and up-regulating Sirt3 [26], or ameliorating myocardial ischemia-reperfusion injury [23]. However, the potential mechanism of polydatin on type-2 diabetes, which is established by high fat feeding and low dose of STZ, is still unclear. The type 2 diabetic model built with high fat diet and STZ is characterized as a non-genetic preclinical type 2 diabetes that reproduces the major metabolic features of insulin resistance and pancreatic β-cell dysfunction, meanwhile mimics the target organ damage in the advanced stage of the disease [30]. Thus, in current study, type 2 diabetic rat is established by combining both high-fat diet and low-dose STZ to explore the protective effects of polydatin against hyperglycemia-induced myocardial injury and oxidative stress. Subsequently, cell modeling is used to investigate the possible targets and underlying mechanisms of polydatin.

## Methods

### Animals

Male Sprague-Dawley rats (200–220 g) were acquired from the Laboratory Animal Center of Xi’an Jiaotong University. The animals had been housed under managed conditions with a 22 ± 2 °C controlled temperature, 50% ± 5% air humidity and 12 h light–12 h dark cycle. The maintenance and treatment of rats were carried out strictly following the guidelines of the Care and Use of Laboratory Animals as published by the US National Academies Press (Eighth Edition, update, 2011). The experimental protocols were approved by the Committee on the Ethics of Animal Experiments of Shaanxi University of Chinese Medicine.

**Fig. 1:**
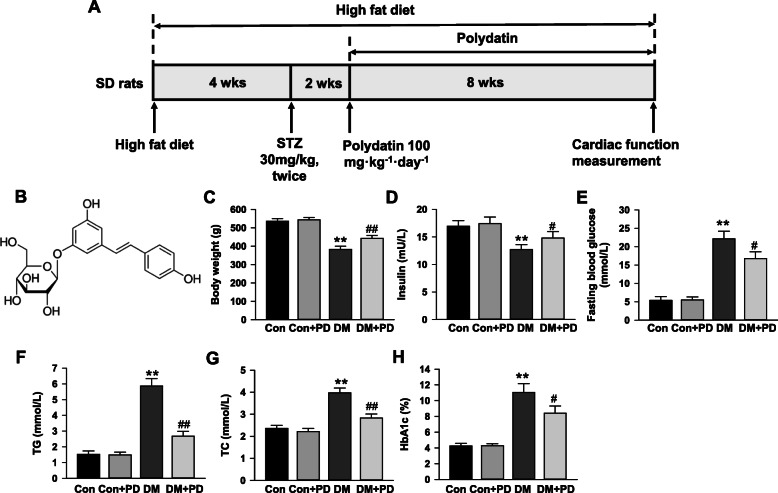
Polydatin improves metabolism abnormalities in diabetic rats. **a** Schematic representation of experimental protocol. Male Sprague–Dawley (SD) rats were fed a high fat diet for 4 weeks and were then injected intraperitoneally with streptozotocin (STZ, 30 mg/kg body weight) for 2 times in 24 h interval. Two weeks after injection, rats with fasting blood glucose levels over 11.1 mmol/L were considered to be diabetes mellitus. The diabetic rats were gavaged with either polydatin (100 mg/kg/day) or equal volume of vehicle for 8 weeks. **b** Chemical structure of polydatin. **c** Body weight of rats in different groups after 8-week polydatin or vehicle treatment. **d** Serum insulin level. **e** Fasting blood glucose level. **f** Serum total triglycerides (TG) level. **g** Serum total cholesterol (TC) level. **h** Glycated hemoglobin (HbA1c) level. Results were expressed as means ± SEM (*n* = 7). *P < 0.05, **P < 0.01 versus Con; *#P <* 0.05, *##P <* 0.01versus DM

The rat model of type 2 diabetes was made by high-fat diet (HFD) and multiple low-dose streptozotocin (STZ), as previously described [30–33]. As shown in Fig. 1a, rats were randomly divided into four groups: the control group (Con, *n* = 7), Con plus polydatin treatment group (Con + PD, n = 7), diabetes mellitus group (DM, *n* = 12), and DM plus PD treatment group (DM + PD, n = 12). Control rats were given a regular diet (kcal%: 10% fat, 70% carbohydrate, and 20% protein; 3.82 kcal/gm, # D12450H, Research Diet, New Brunswick, NJ, USA). Other rats received a HFD (kcal%: 45% fat, 35% carbohydrate, and 20% protein; 4.70 kcal/gm, #D12451, Research Diet, New Brunswick, NJ, USA) for 4 weeks, then two intraperitoneal injections of STZ (Sigma-Aldrich, USA, 30 mg/kg body weight, dissolved in citrate buffer—pH 4.5) were performed in 24 h interval. Control rats received the vehicle (citrate buffer) at the same time. At the 14 days after the first STZ injection, fasting blood glucose (FBG) were detected with a glucometer (One-Touch, Johnson and Johnson, USA) in caudal vein blood. The model was successfully established if FBG levels exceed 11.1 mmol/L [30–33]. Rats in DM group were removed from the experiment when FGB did not reach these glucose values, and FGB had been weekly measured to make certain that diabetes was not reversed. After confirmation of diabetes, rats were divided in 2 groups containing 7 in each. Then, the rats in Con + PD and DM + PD group were administered with polydatin (100 mg/kg, dissolved in 0.5% carboxymethylcellulose) for 8 weeks, while the Con and DM group were given the same volume of vehicle throughout the experimental period. The rats in all groups were treated 6 days every week by gavage, and allowed free access to the diet in the following 8 weeks. Polydatin (3,4′,5-trihydroxystilbene-3 -b-D-glucopyranoside; wt. 390.38; Fig. 1b) was provided by Sigma-Aldrich (USA). The dosages of polydatin (100 mg/kg/day) used in vivo was chosen based on previous reports showing that polydatin at this dose resulted in a better outcome in improving lipid and glucose metabolism in experimental diabetes [34–37]. After 8 weeks of treatment and feed, cardiac function was investigated by echocardiography, and then blood was collected from the abdominal aorta for biochemical assay after fasting 12 h under anesthesia with 3% isoflurane inhalation, which resulted in animals’ death because of bleeding shock. Part of the heart was preserved in formaldehyde for histological analysis and the others was immediately processed for protein and RNA extraction.

### Biochemical assay

After 8-weeks polydatin administration, One-Touch glucometer (Johnson and Johnson, USA) was used to determine the FBG in the caudal vein blood. Diagnostic kits (Jiancheng Biotech, Nanjing, China) were used to measure glycosylated hemoglobin (HbA1c) in plasma. Colorimetric assay kits according to the standard methods (Jiancheng Biotech, Nanjing, China) were used to detect the total cholesterol and triacylglycerol levels. ELISA assay kit from RayBiotech (#P01323, Norcross, GA, USA) was used to evaluate the insulin levels.

### Cardiac function measurement

Echocardiography was carried out by Philips 7500 (Amsterdam, NL), as previously described [16]. Under anesthesia with 2% isoflurane inhalation, the 15-MHz transducer was placed in a left lateral decubitus position, and M type ultrasound was applied to obtain the left ventricular posterior wall thickness (LVPWD), left ventricular ejection fraction (LVEF), left ventricular end-diastolic diameter (LVEDD) and left ventricular shortening fraction (LVFS). The early (E) and late (A) diastolic filling velocities of the apical four chambers and the ratio of E/A was evaluated by a pulsed Doppler method.

### Histological analysis

The rat heart was embedded in paraffin after fixation with 10% formaldehyde. Five sub-serial sections with 10 μm thickness at 0.3 mm intervals was obtained from the middle part of the heart. Myocyte cross-sectional area was determined by hematoxylin and eosin staining, and interstitial fibrosis in transverse sections was examined by picrosirius red staining as previously described [27]. The micrographs were analyzed by Image-Pro plus 6.0 (Media Cybernetics, Bethesda, MD). In each tissue section, the cross-sectional area was assessed in 40 myocytes from 10 random fields. The interstitial fibrosis was calculated by the picrosirius red stained area divided by total myocardial area from 10 random fields [38]. All morphometric measurements were performed in a blinded manner.

### Detection of ROS level

ROS level in myocardial tissue was examined by dihydroethidium (DHE) staining as previously described [16]. The left ventricular myocardium was cut into 20-μm sections in a cryostat, which were incubated with DHE (2 μmol/L; Molecular Probes, D-11347) at 37 °C for 1 h under dark conditions. Fluorescence images were checked with a fluorescent microscope (BX-53, Olympus Optical, Tokyo, Japan). For quantification, 8 tissue sections were taken from each animal and collected as the average value of each experimental condition.

### Detection of NADPH oxidase activity

The NADPH oxidase activity was determined by lucigenin chemiluminescence’s assay as previously described [27]. Left ventricular lysates or cultured cells were prepared in lysis buffer (20 mmol/L KH2PO4, 1 mmol/L phenylmethylsulfonyl fluoride, and 1 mmol/L EDTA, pH 7.0) as described above. After centrifugated at 3000 g for 10 min, the supernatant was placed into a 96-well microplate (100 mg/well). Then the samples were incubated with 5 mmol/L lucigenin for 2 min, and 100 mmol/L NADPH was added as substrate. Luminometer (Wallac 1450, PerkinElmer Life and Analytical Sciences, Waltham, MA) was used to examine the chemiluminescent signal every 15 s for 3 min. The superoxide anion produced in the reaction were determined as the NADPH oxidase activity. The protein concentrations of samples were measured by a protein quantification kit (Bio-Rad, Hercules, CA, USA). Data were expressed as mmol/mg of protein per minute.

### Cell culture

Embryonic rat cardiac H9c2 cells were provided by Costar Corning Inc. (Corning, CA, USA) and cultured in DMEM Medium with 5.5 mmol/L glucose (normal glucose) added with 10% fetal bovine serum in a 5% CO_2_ humidified atmosphere at 37 °C. For each experiment, H9c2 cells were plated and were grown to reach 70–80% confluence for 24 h.

### Cell viability and lactate dehydrogenase (LDH) activity assay

The viability of cells was measured by the cell counting kit–8 (CCK-8) assay kit (Dojindo, Kumamoto, Japan). In brief, H9c2 cells were cultured in 96-well plates at a density of 1 × 10^4^ cells. After incubation for 12 h in serum-free medium, the H9c2 cells were treated with DMEM including 5.5 mmol/L glucose (Control, Con), Con plus 10 μmol/L polydatin, Con + 20 μmol/L polydatin, Con + 40 μmol/L polydatin, 30 mmol/L glucose (High glucose, HG), HG + 10 μmol/L polydatin, HG + 20 μmol/L polydatin, and HG + 40 μmol/L polydatin. Following incubation for 24 h, cells were washed with PBS and cultured with 10 μmol CCK-8 for 2 h. A microplate reader (Bio-Tek Instruments, Winooski, VT, USA) was used to measure the absorbance at 450 nm.

LDH assay kit (Jiancheng, Nanjing, China) was applied to check the LDH activity in the supernatants according to the manufacturer’s protocol.

### Intracellular ROS detection

Intracellular ROS in H9c2 cells was determined by the fluorescent indicator 20,70-dichlorofluorescein diacetate (DCF-DA; Sigma) as described previously [27]. Cells were cultured in 96-well plates, and incubated in a light-protected chamber with 5 mmol/L DCF-DA solution at 37 °C for 30 min. The fluorescence was determined by a fluorescent plate reader (Spectra MAX GeminiXPS, Molecular Devices, CA, USA) at 485 nm for excitation and 530 nm for emission.

### ELISA assay

The levels of interleukin (IL)-1β, IL-6, tumor necrosis factor (TNF)-α and vascular cell adhesion molecule 1 (VCAM-1) in heart homogenates and cultured H9c2 cells were detected by ELISA kits (IL-1β: ab100768; IL-6: ab100772; TNF-α: ab46070; Abcam Inc., Cambridge, MA, USA; VCAM-1: E07275r, cusabio, Wuhan, China). Cytokines levels were expressed in ng/mg protein.

### Real-time PCR

The real-time PCR was conducted as described by us previously [27, 28]. A RNeasy kit (Qiagen, Valencia, CA, United States) was applied to extract the total RNA from cultured H9c2 cells or left ventricular lysates. TaqMan PCR probes for rat TNF-α (Rn99999017_m1), IL-1β (Rn00580432_m1), IL-6 (Rn01410330_m1) and VCAM-1(Rn00563627_m1), NOX4(Rn00585380_m1), NOX2(Rn00576710_m1), and NOX1(Rn00586652_m1) were provided by Thermo Fisher Scientific (Carlsbad, CA, USA). An ABI PRISM 7500 system (Applied Biosystems, Foster, CA, USA) was used to detect the amplification by a comparative cycle of threshold (CT) fluorescence method. The data were shown as the ratio of the mRNA of interest to 18S rRNA.

### Western blot analysis

The western blot experiment was performed as described by us previously [27, 28]. Left ventricular or H9c2 cell samples were placed in an ice-cold lysing buffer. After samples homogenization, the protein concentration was determined by a protein quantification kit (Bio-Rad, Hercules, CA, USA). The wells of SDS-PAGE gel were loaded with 25 μg of total protein from the samples. After running the gel, the protein was transferred to the nitrocellulose membrane (Millipore, Billerica, MA, United States), which were blocked with blocking buffer for 1 h. Then the primary antibody was added and incubated overnight at 4 °C, including NOX4 (ab154244, Abcam, Cambridge, United Kingdom), NOX2 (ab131083, Abcam, Cambridge, United Kingdom), NOX1 (ab131088, Abcam, Cambridge, United Kingdom), p65 (ab16502, Abcam, Cambridge, United Kingdom), Histone (ab1791, Abcam, Cambridge, United Kingdom), phosphorylation IκB-α (sc-52,943, Santa Cruz Biotechnology, CA, USA), or IκB-α (sc-1643, Santa Cruz Biotechnology, CA, USA). After three washes with PBST, conjugated secondary antibody (Bio-Rad, Hercules, CA, USA) was added for 1 h, and then the membrane was treated with chemiluminescence substrate (Amersham Pharmacia Biotechnology, Piscataway, NJ, United States). The blot signal was analyzed by Quantity One Software (Bio-Rad, Hercules, CA, USA).

### Statistical analysis

Results are expressed as mean ± SEM. Statistical comparisons were made by one-way ANOVA followed by Newman-Keuls post-hoc analysis using Sigmaplot 12 software (San Jose, CA). *P <* 0.05 was considered as statistically significant.

## Results

### Polydatin reduced the metabolism abnormalities

The results of the metabolic characteristics of the animals (Fig. 1c-h) show that compared with the control, the fasting blood glucose, total cholesterol and triglycerides, and glycated hemoglobin were markedly (*P <* 0.05) raised in diabetic rats induced by HFD and multiple low-dose STZ, while body weight and fasting insulin were reduced, which were significantly (*P <* 0.05) improved by eight-weeks polydatin treatment. All of above metabolic parameters in non-diabetic rats were not altered by polydatin.

### Polydatin improved cardiac dysfunction in diabetic rats

**Fig. 2:**
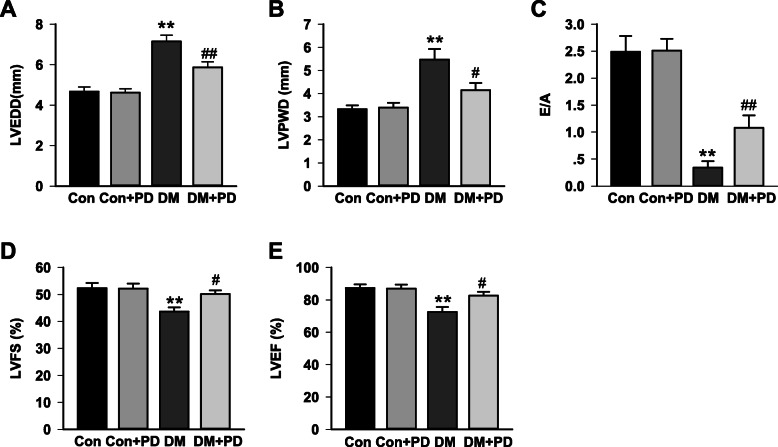
Polydatin attenuates diabetes-induced left ventricular dysfunction. Echocardiography was performed on control or diabetic rats treated with either polydatin (90 mg/kg/day) or vehicle for 8 weeks. Panel (**a**) Left ventricular end diastolic diameter (LVEDD); (**b**) Left ventricular posterior wall thickness (LVPWD); (**c**) The ratio of E/A; (**d**) Left ventricular shortening fraction (LVFS); (**e**) Left ventricular ejection fraction (LVEF). Data were expressed as means ± SEM (n = 7). *P < 0.05, **P < 0.01 versus Con; *#P <* 0.05, *##P <* 0.01versus DM

Cardiac performance parameters derived from echocardiography are shown in Fig. 2. The diabetic rats induced by HFD and multiple low-dose STZ showed a significant cardiac dysfunction, as shown by increased LVEDD (7.15 ± 0.31 mm in DM group vs. 4.67 ± 0.23 mm in Con group) and LVPWD (5.47 ± 0.46 mm in DM group vs. 3.32 ± 0.17 mm in Con group), and reduced E/A ratio (0.34 ± 0.12 in DM group vs. 2.49 ± 0.29 in Con group), reduced LVEF (72.53 ± 3.07% in DM group vs. 87.32 ± 2.23% in Con group) and LVFS (43.63 ± 1.63% in DM group vs. 52.38 ± 1.93% in Con group) compared with that in control group. Polydatin treatment significantly attenuated the cardiac diastolic and systolic dysfunction with improved E/A ratio(1.08 ± 0.23 in DM + PD group vs. 0.34 ± 0.12 in DM group), LVEF (82.59 ± 2.36% in DM + PD group vs.72.53 ± 3.07% in DM group) and LVFS (50.16 ± 1.32 in DM + PD group vs. 43.63 ± 1.63% in DM group) and decreased LVEDD (5.87 ± 0.27 mm in DM + PD group vs.7.15 ± 0.31 mm in DM group) and LVPWD (4.15 ± 0.31 mm in DM + PD group vs. 5.47 ± 0.46 mm in DM group).

### Polydatin attenuates cardiac hypertrophy and interstitial fibrosis

**Fig. 3:**
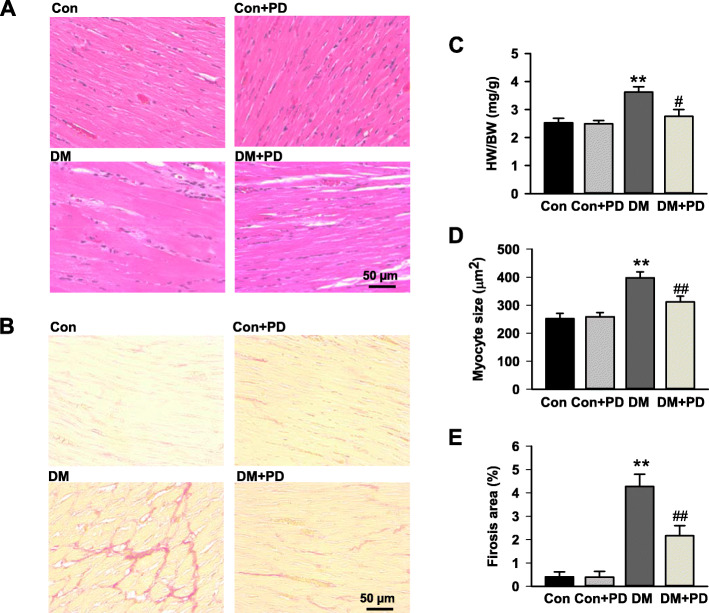
Polydatin attenuates diabetes-induced cardiac remolding. **a** Representative cross-sections of rat left ventricles histochemically stained with H&E staining. **b** Representative images of myocardium with cardiac fibrosis with Sirius red staining. **c** Heart weight-to-body weight ratio (HW/BW) of rats in different groups after 8-week polydatin or vehicle treatment. **d** Bar graph shows quantitative analysis of cardiomyocyte cross-sectional areas. **e** Bar graph shows quantified interstitial fibrotic areas (%). The scale in the images is 50 μm. Magnification × 200. Results were expressed as means ± SEM (n = 7). *P < 0.05, **P < 0.01 versus Con; *#P <* 0.05, *##P <* 0.01versus DM

As shown in Fig. 3, the HW/BW and cardiomyocyte size in diabetic rats markedly (*P* < 0.05) increased compared with that in control rats. Polydatin treatment (100 mg/kg/d, 8 weeks) markedly attenuated diabetes-associated rises in HW/BW by 29.8 ± 2.5% (Fig. 3c), and the cardiomyocyte size by 31.5 ± 2.4% (Fig. 3d). Additionally, polydatin significantly (P < 0.05) attenuated the increase in interstitial fibrosis stained with Sirius Red by 58.3 ± 3.2% in the diabetic hearts (Fig. 3b, e).

### Polydatin inhibits myocardial oxidative stress

**Fig. 4:**
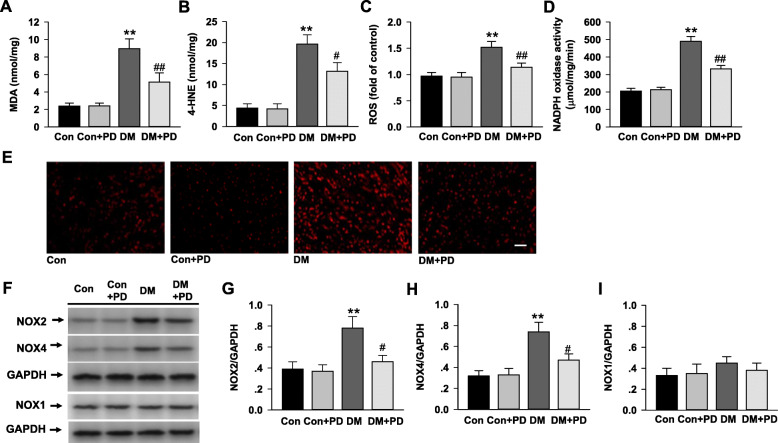
Polydatin attenuates diabetes-induced myocardial oxidative stress and NADPH oxidase activation. **a** Malondialdehyde (MDA) levels in the myocardial tissues were determined using colorimetric assay kits. **b** 4-HNE levels in the myocardial tissues determined by ELISA. **c** Reactive oxygen species (ROS) levels in myocardium detected by DHE staining. **d** NADPH oxidase activity measured in myocardium by lucigenin chemiluminescence. **e** Representative images of DHE staining for detecting ROS in myocardium. **f**-**i** The protein expression of NOX2, NOX4, and NOX1 were detected by Western blotting. Results were expressed as means ± SEM (n = 7). *P < 0.05, **P < 0.01 versus Con; *#P <* 0.05, *##P <* 0.01versus DM

The diabetic rats displayed a remarkable increase in MDA (Fig. 4a; *P* < 0.01), 4-HNE (Fig. 4b; *P <* 0.01) and ROS generation of the heart tissue (Fig. 4c, d and e; *P <* 0.01) compared with that of normal rats. The heart of diabetic rats received 8-weeks polydatin demonstrated a significant reduction in contents of MDA and 4-HNE, and cardiac ROS production.

NADPH oxidase activity was significantly elevated in the diabetic heart, which was reversed by polydatin treatment. In line with the activities, the protein levels of NOX2 and NOX4 were also markedly increased in the diabetic heart (Fig. 4f, g, and h), and polydatin treatment significantly reduced the expression of NOX2 and NOX4 in the myocardium of diabetic rats. The NOX1 level had no change in all rats (Fig. 4i).

### Polydatin suppresses myocardial nuclear factor-κB (NF-κB) and inflammation

**Fig. 5:**
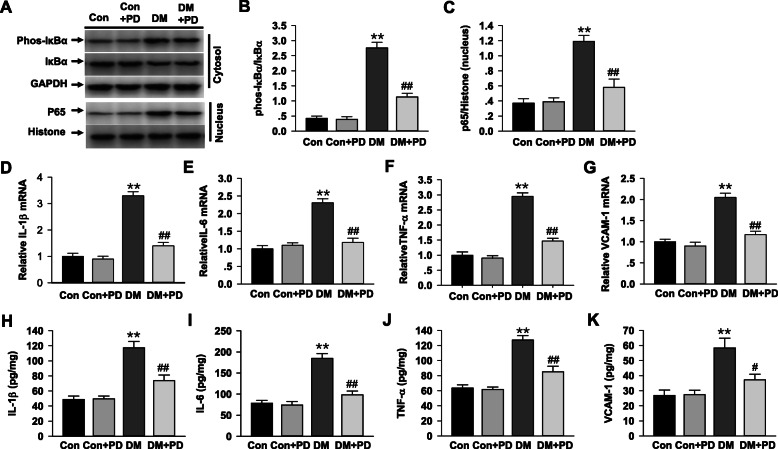
Polydatin attenuates diabetes-induced myocardial NF-κB activation. **a**-**c** Western blot analysis of I-κB-α expression and its phosphorylation in the cytosol fractions, and nuclear translocation of p65 in the nuclear fraction of the heart tissue in different groups after respective treatments. **d**-**g** Real-time PCR analysis of IL-1β, IL-6, TNF-α and VCAM-1 mRNA level in each group of rats. **h**-**k** The expression of IL-1β, IL-6, TNF-α and VCAM-1 was determined by ELISA as described in Methods. Results were expressed as means ± SEM (n = 7). *P < 0.05, **P < 0.01 versus Con; *#P <* 0.05, *##P <* 0.01versus DM

As an indicator of the NF-κB activation, phosphorylated IκBα in the cytosolic fraction was markedly increased (Fig. 5a and b, *P <* 0.05) in the diabetic heart, a subunit of the NF-kB transcription factor complex, nuclear translocation of p65 also found elevated in the nuclear fraction (Fig. 5c, *P <* 0.05), both of them were significantly suppressed by polydatin (Fig. 5a-c). Besides, the polydatin treatment significantly dampened (*P <* 0.05) the increased NF-κB-dependent mRNA and protein levels of IL-1β (Fig. 5d and h), IL-6 (Fig. 5e and i), TNF-α (Fig. 5f and j) and VCAM-1(Fig. 5g and k) in the diabetic myocardial tissues.

### Polydatin inhibits NADPH oxidase activity in HG-induced H9c2 cells

**Fig. 6:**
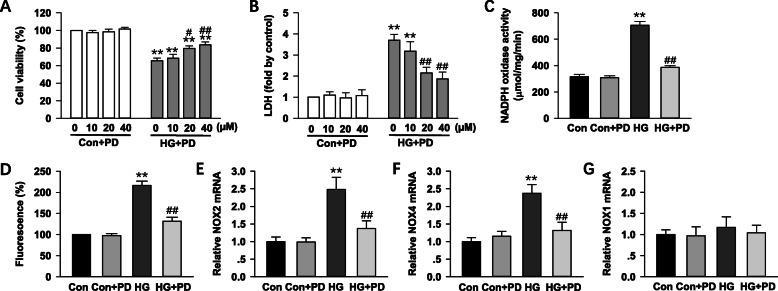
Polydatin prevents NADPH oxidase activity in HG-induced H9c2 cells. H9c2 cells were cultured in DMEM containing 30 mmol/L glucose (HG) or 5.5 mmol/L glucose (normal glucose) and treated with polydatin (0–40 μmol/L) for 24 h. **a** Cell viability was measured by a Cell Counting Kit-8 assay. **b** Cytotoxicity was detected via lactate dehydrogenase (LDH) assay. **c** NADPH oxidase activity was measured by lucigenin chemiluminescence’s assay. **d** Intracellular superoxide anion levels were detected in H9c2 cells using fluorogenic probe DCFH-DA. **e**, **f** and **g** Summary of real-time PCR data showing levels of NOX2, NOX4, and NOX1 mRNA in H9c2 cells. Data were expressed as means ± SEM (*n* = 6–7). *P < 0.05, **P < 0.01 versus control values; *#P <* 0.05, *##P <* 0.01versus HG

To understand the direct effect of polydatin on HG -stimulated myocyte injury, H9c2 cells were cultured in DMEM with 5.5 mmol/L glucose (normal glucose) or 30 mmol/L glucose (HG), and then treated with polydatin (0–40 μmol/L) for 24 h. As shown in Fig. 6a and b, the cell viability and LDH release assay revealed that polydatin treatment (0–40 μmol/L) dose-dependently suppressed HG-induced proliferation and injury of H9c2 cardiomyocyte. The 40 μmol/L of the polydatin was used in the following experiments based on the above findings that shows its significant effect on the cell’s proliferation and injury.

NADPH oxidase activation in H9c2 cells cultured in HG was further evaluated. The activities of NADPH oxidase in the presence of HG was markedly raised to 224 ± 11% of control, which was blunted by polydatin treatment to 125 ± 6% of control (*P <* 0.05). Polydatin had no effect on the NADPH oxidase activity in control cells. The DCF-DA fluorescence assay also confirmed that ROS production in H9c2 cell was found increased by HG, which was suppressed by polydatin (Fig. 6c and d). In addition, the effects of polydatin treatment on NADPH oxidase subunits mRNA levels are demonstrated in Fig. 6e, f, and g. Polydatin reduced the elevated mRNA levels of NOX2 and NOX4 in HG-induced H9c2 cells, which indicated that polydatin reduced NADPH oxidase activation in cardiomyocyte.

### Polydatin alleviates NF-κB activation in HG-induced H9c2 cells

**Fig. 7:**
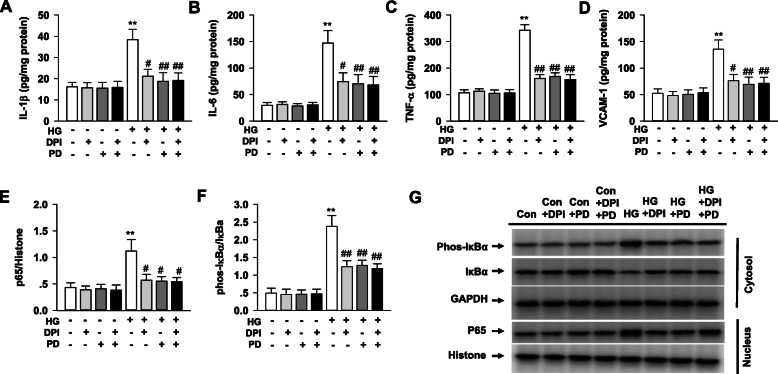
Polydatin inhibits NF-κB activation in HG-induced H9c2 cells. H9c2 cells were cultured in DMEM containing 30 mmol/L glucose (HG) or 5.5 mmol/L glucose (normal glucose) and treated with polydatin (40 μmol/L), DPI (a NADPH oxidase inhibitor, 10 μmol/L) or polydatin (40 μmol/L) + DPI (10 μmol/L) for 24 h. The expression of TNF-α, IL-1β, IL-6 and VCAM-1 was determined by ELISA. NF-κB expression was assessed by Western blot. **a** IL-1β levels in H9c2 cells. **b** IL-6 levels in H9c2 cells. **c** TNF-α levels in H9c2 cells. **d** VCAM-1 levels in H9c2 cells. **e** The expression of p65 in nucleus of H9c2 cells. **f** The expression of phosphorylation of IκB-α in cytoplasm of H9c2 cells. **g** Representative immunoblots of p65 and phosphorylation of IκB-α protein levels in H9c2 cells. Results were expressed as means ± SEM (n = 6). *P < 0.05, **P < 0.01 versus control values; *#P <* 0.05, *##P <* 0.01versus HG

As shown in Fig. 7, the HG stimulation in H9c2 cells displayed an increase of IL-1β, IL-6, TNF-α and VCAM-1, as well as NF-κB activation indicated by the increased phosphorylation of IκB-α in the cytoplasm and elevated p65 in nucleus, which were all inhibited by polydatin treatment. Inhibition of NADPH oxidase with diphenyleneiodonium (DPI) also showed similar effects with polydatin in attenuating the NF-κB activation and inflammatory cytokine generation in HG-induced H9c2 cells. Furthermore, compared with DPI alone, treatment with DPI plus polydatin did not increase the intervention effect of polydatin on the NF-κB activation and cytokine generation in HG-induced H9c2 cells.

## Discussion

This study provides the direct evidence that polydatin protects the heart from myocardial injury in diabetic rats induced by HFD and low-dose of STZ. Our data indicates that cardiac dysfunction as well as cardiac hypertrophy and interstitial fibrosis has been significantly improved by polydatin in diabetic rats. Further study has identified that reduction of myocardial oxidative stress, as well as decreased activation of myocardial NF-κB and inflammation, were associated with the cardioprotective effect of polydatin.

Type 2 diabetic model which induced by HFD and low-dose STZ has been viewed as an ideal diabetic model [39, 40]. This model is built to test the antidiabetic agents by mimicking the metabolic and pathological features of type 2 diabetes [41]. In present study, diabetic model induced by HFD and low-dose STZ displays the raise in fasting blood glucose, glycated hemoglobin, and ratio of heart weight to body weight, as well as decreased fasting serum insulin level and ventricular function. These results confirm the successfully established diabetic model with cardiomyopathy that is consistent with previous studies [16, 31].

Polydatin is a resveratrol glycoside extracted from a Chinese herb called *Polygonum cuspidatum* that has been applied extensively in China to treat a variety disease including diabetes [21]. Current study shows that polydatin reduces the level of hyperglycemia and hyperlipemia, also ameliorates insulin resistance in diabetic rats, which is consistent with the results of others [34, 35]. The pivotal finding in our study is that 8-weeks polydatin treatment significantly improved the diastolic and systolic dysfunction, attenuated hypertrophy and interstitial fibrosis in the diabetic hearts, suggesting myocardial protection of polydatin in diabetes. Evidence from preclinical trials has suggested that polydatin protects cardiomyocytes from ischemia and ischemia-reperfusion injury and heart failure in non-diabetic models [22, 23]. It is worth noting that the anti-diabetic functions of polydatin contribute to its cardioprotection, while polydatin may also exert direct protecting against diabetic heart. This is possible because the tissue distribution of oral polydatin administration is more than that in blood, meanwhile the heart was one of the most strongly distributed organs [21]. Our data from the in vitro experiment confirm the in vivo findings that polydatin performed a direct action against high glucose-induced cardiomyocyte injury. Furthermore, the relatively limited hypoglycemic effect is supported by higher fasting glucose in the therapeutic group than control group. All above indicates that the protective effects against diabetic heart might be more important besides the slight hypoglycemic activities of polydatin in treatment of diabetes.

Excessive oxidation leads to ROS aggregation, subsequently oxidative stress plays a pivotal role in the development of diabetes-related complications [5, 42, 43]. Through current study, we have observed that the increased production of MDA, 4-HNE, and ROS are significantly reduced by polydatin treatment. It agrees with the suppressive function of polydatin on the hyperglycemia-induced ROS accumulation which is conducted in H9c2 cells. However, the production of ROS at baseline hasn’t been changed by using polydatin alone, the reason may lie in the basal oxidative activity was kept efficiently to a minimum in the physiological state by superoxide dismutase and other endogenous ROS scavengers. The findings in the current study indicate that when the hyperglycemia-induced superoxide generation blocked by polydatin, myocardial injury also alleviated, which strongly supports the notion that diabetic cardiomyopathy is mainly mediated by oxidative stress.

For the heart tissues, NADPH oxidase exists as the predominant ROS source [4, 44]. Several lines of evidence have demonstrated that the NADPH oxidases (including NOX2 and NOX4) are directly enhanced by the hyperglycemia in diabetic heart [11–14], indicating that ROS derived from NADPH oxidase might contribute to diabetic myocardial damage. The increase of antioxidant enzymes and the decrease of redox-sensitive transcription factors have been considered as the main antioxidant activity of polydatin [45, 46]. The present study shows polydatin significantly reduced the high level of ROS, as well as the NOX2 and NOX4 expression in the diabetic heart. The suppressive effect suggests that polydatin has huge possibility to attenuate superoxide production through inhibiting NADPH oxidase, also highlights that the polydatin has antioxidant property by inhibiting NADPH oxidase directly.

A maladaptive pro-inflammatory response has also been implicated during the developing of diabetic cardiomyopathy. Activation of TNF-α, IL-6, IL-1β and VCAM-1, are all involved in the oxidative stress, cardiac remodeling, and diastolic dysfunction of heart [10, 19]. As a key transcription factor, NF-κB controls multiple target genes and is primarily involved in inflammation [17]. In diabetes, ROS can directly induce NF-κB activation, thus promoting the release of inflammatory cytokines [19, 47]. Additionally, NF-κB and ROS provoke each other into a vicious cycle, aggravating myocardial damage [15, 18]. In line with the above studies, our present study shows that the expressions of IL-1β, IL-6, TNF-α and VCAM-1 are upregulated by the increased phosphorylation of IκB-α (NF-κB activation) in cytoplasm and elevated p65 in nucleus in the diabetic heart tissue. All these alterations are significantly attenuated by polydatin in the heart tissues from diabetic rats and HG-induced H9c2 cells, which indicate that the polydatin might reduce inflammation through decreasing the NF-κB activities and the inflammatory cytokines production. This notion is confirmed by the HG-induced ROS generation, NF-κB activation and inflammatory cytokines production are all attenuated by inhibiting NADPH oxidase with DPI in H9c2 cells. Therefore, NF-κB pathway exhibits an important feature to mediate the hyperglycemia-induced inflammation via NADPH oxidase-derived ROS generation in diabetic heart.

One concern is the possible mechanism of correlation with NADPH oxidase and NF-κB during the developing of diabetes-related cardiomyopathy. It has been reported that ROS, which is produced by up-regulating NADPH oxidase, plays a key role in structural-functional alterations of the diabetic heart [4]. Evidences indicated that ROS cause the activating of NF-κB mainly by inhibiting the phosphorylation of IκBs [10, 48–50]. Recently, a study demonstrates that histone deacetylase makes a contribution to the modulation of NADPH oxidase expression and the ROS generation in diabetes models [51]. Zarzuelo’ study indicated that impaired activity of SIRT1 up-regulated NADPH oxidase-derived ROS production and induced endothelial dysfunction in the vascular wall [52]. Bagul et al. also reported that in diabetic heart, SIRT1 activated by resveratrol results in deacetylation of NF-κB p65 and H3, therefore reduces transcription of NADPH oxidase and attenuates cardiac oxidative stress and dysfunction [53]. In addition, it has been suggested polydatin increased SIRT1 activity leading to attenuation of mitochondrial damage in hemorrhagic shock [54]. Thus, it is probable that the cardioprotective effects of polydatin by inhibiting NADPH oxidase and NF-κB activities in diabetic heart may be mediated by upregulating SIRT1. Despite these previous reports, correlation with SIRT1, NADPH oxidase and NF-κB in diabetic cardiomyopathy has not been fully clear. For the purpose to clarify the underlying mechanisms revealing cardioprotective effects of polydatin, it is necessary to confirm the key signaling molecules participating the development of diabetic cardiomyopathy, as well as the cross talk between them.

## Conclusion

Our study indicates that polydatin alleviated diabetes-associated diastolic dysfunction, cardiac remolding, myocardial oxidative stress, and cardiac inflammation. The cardioprotective effects of polydatin might be mediate through its inhibition of NADPH oxidase and NF-κB activation. These finding suggests the potential therapeutic prospect of polydatin for the intervention on diabetic-associated cardiovascular complications. Because of no specific drugs for the diabetic cardiomyopathy treatment and the adverse effects involved, polydatin may be a promising natural agent for disrupting the pathogenesis of diabetic cardiomyopathy.

## Supplementary Information

**Additional file 1.**

## Data Availability

All data generated or analyzed during this study are included in this published article.

## Abbreviations

Con: Control
CT: Cycle of threshold
DHE: Dihydroethidium
DCF-DA: 20,70-dichlorofluorescein diacetate
DM: Diabetes mellitus
DPI: Diphenyleneiodonium
FBG: Fasting blood glucose
HFD: High-fat diet
HbA1c: Glycosylated hemoglobin
HG: High glucose
4-HNE: 4-hydroxynonenal
IL: Interleukin
LDH: Lactate dehydrogenase
LVEDD: Left ventricular end diastolic diameter
LVPWD: Left ventricular posterior wall thickness
LVFS: Left ventricular shortening fraction
LVEF: Left ventricular ejection fraction
MDA: Malondialdehyde
NADPH: Nicotinamide adenine dinucleotide phosphate
Nox: NADPH oxidase
NF-κB: Nuclear factor-κB
PD: Polydatin
ROS: Reactive oxygen
STZ: Streptozotocin
TNF: Tumor necrosis factor
VCAM-1: Vascular cell adhesion molecule 1
SIRT1: Silent information regulator 1
