# Supplementary material for: Cardioprotective effects of polydatin against myocardial injury in diabetic rats via inhibition of NADPH oxidase and NF-κB activities
Source: BMC Complement Med Ther. 2020 Dec 11;20:378. doi: 10.1186/s12906-020-03177-y (PMC7733248; doi:10.1186/s12906-020-03177-y)

Original uncropped western-blot images

Figure 4F: NOX2 protein. Western blot was performed on the hearts of control or diabetic rats treated with either polydatin (90 mg/kg/day) or vehicle for 8 weeks. 1st—4th lane: CON, CON+PD, DM, DM+PD. Observed band size: 67 kDa.

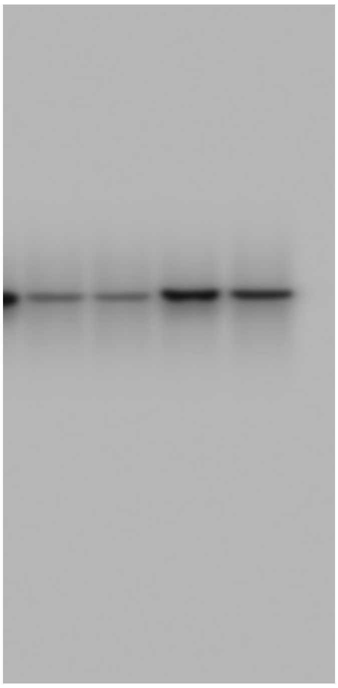

Figure 4F: NOX4 protein. Western blot was performed on the hearts of control or diabetic rats treated with either polydatin (90 mg/kg/day) or vehicle for 8 weeks. 1st—4th lane: CON, CON+PD, DM, DM+PD. Observed band size: 65 kDa.

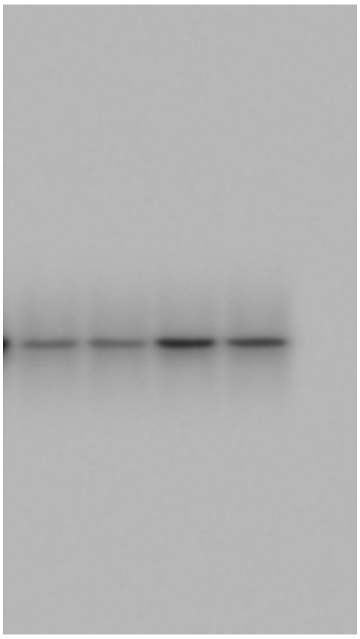

Figure 4F: NOX1 protein. Western blot was performed on the hearts of control or diabetic rats treated with either polydatin (90 mg/kg/day) or vehicle for 8 weeks. 1st—4th lane: CON, CON+PD, DM, DM+PD. Observed band size: 65 kDa.

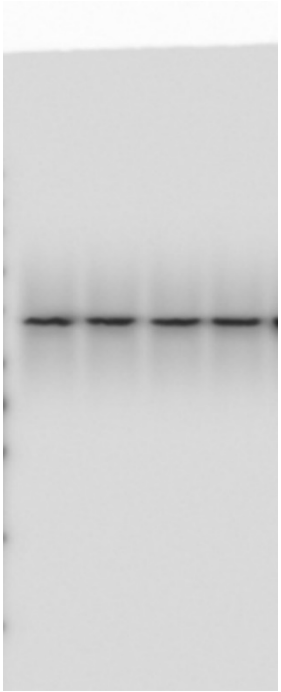

Figure 4F: GAPDH protein. Western blot was performed on the hearts of control or diabetic rats treated with either polydatin (90 mg/kg/day) or vehicle for 8 weeks. 1st—4th lane: CON, CON+PD, DM, DM+PD. Observed band size: 40 kDa.

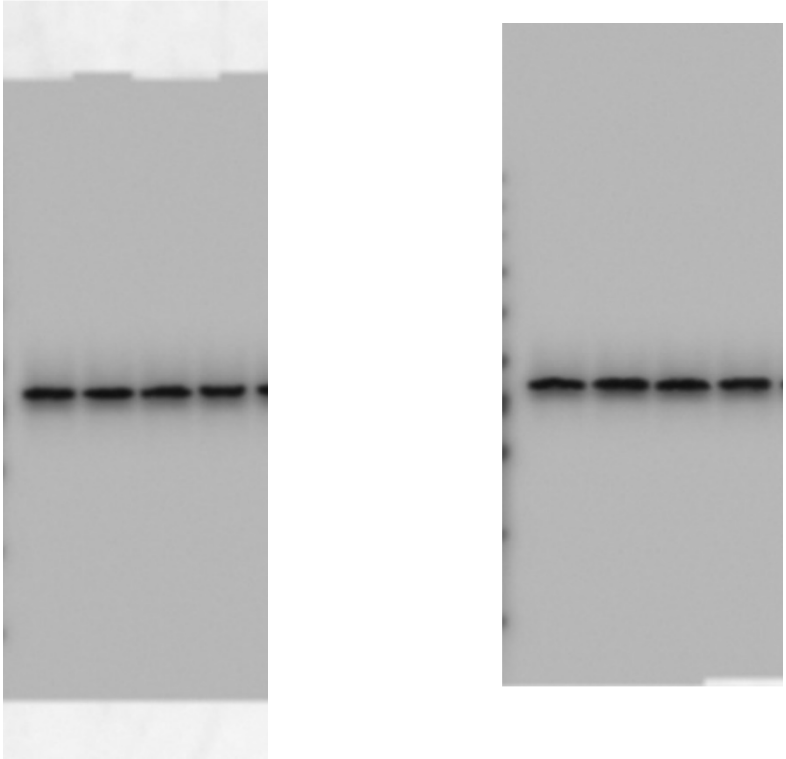

Figure 5A: Phosphorylation of IκB-α protein. Western blot was performed on the hearts of control or diabetic rats treated with either polydatin (90 mg/kg/day) or vehicle for 8 weeks. 1st—4th lane: CON, CON+PD, DM, DM+PD. Observed band size: 85 kDa.

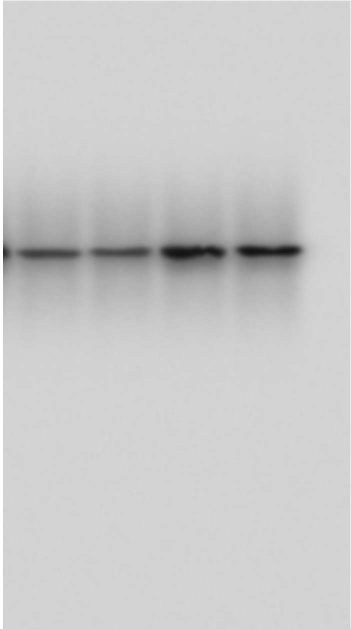

Figure 5A: IκB-α protein. Western blot was performed on the hearts of control or diabetic rats treated with either polydatin (90 mg/kg/day) or vehicle for 8 weeks. 1st—4th lane: CON, CON+PD, DM, DM+PD. Observed band size: 85 kDa.

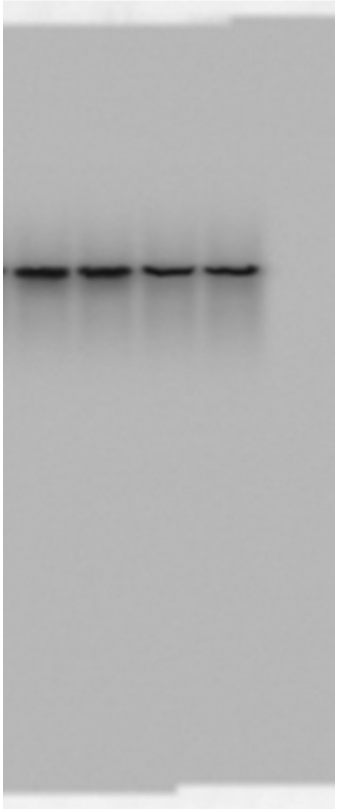

Figure 5A: GAPDH protein. Western blot was performed on the hearts of control or diabetic rats treated with either polydatin (90 mg/kg/day) or vehicle for 8 weeks. 1st—4th lane: CON, CON+PD, DM, DM+PD. Observed band size: 65 kDa.

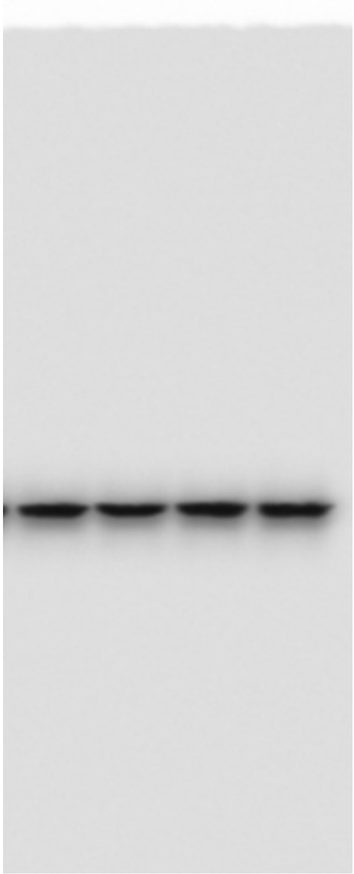

Figure 5A: p65 protein. Western blot was performed on the hearts of control or diabetic rats treated with either polydatin (90 mg/kg/day) or vehicle for 8 weeks. 1st—4th lane: CON, CON+PD, DM, DM+PD. Observed band size: 65 kDa.

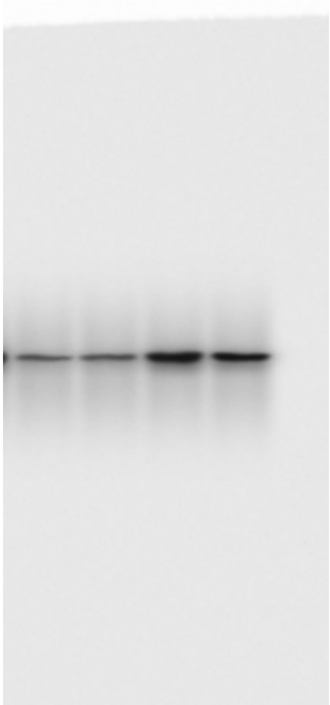

Figure 5A: Histone protein. Western blot was performed on the hearts of control or diabetic rats treated with either polydatin (90 mg/kg/day) or vehicle for 8 weeks. 1st—4th lane: CON, CON+PD, DM, DM+PD. Observed band size: 15 kDa.

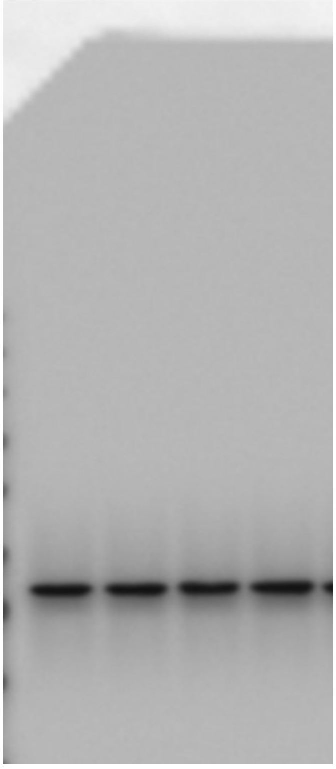

Figure 7G: Phosphorylation of IκB-α protein in the cytoplasm of H9c2 cells. H9c2 cells were cultured in DMEM containing 30 mmol/L glucose (HG) or 5.5 mmol/L glucose (normal glucose) and treated with polydatin (40 μmol/L), DPI (a NADPH oxidase inhibitor, 10μmol/L) or polydatin (40 μmol/L) +DPI (10μmol/L) for 24 h. 1st—4th lane: CON,CON+DPI,CON+PD CON+DPI+PD; 5th—8th lane: HG,HG+DPI,HG+PD HG+DPI+PD. Observed band size: 85kDa.

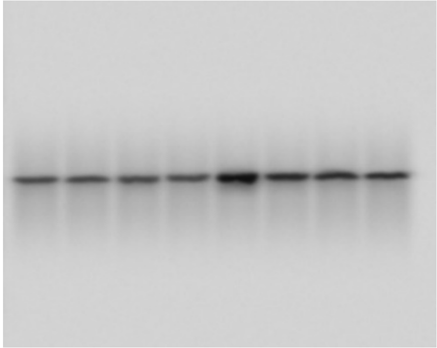

Figure 7G: IκB-α protein in the cytoplasm of H9c2 cells. H9c2 cells were cultured in DMEM containing 30 mmol/L glucose (HG) or 5.5 mmol/L glucose (normal glucose) and treated with polydatin (40 μmol/L), DPI (a NADPH oxidase inhibitor, 10μmol/L) or polydatin (40 μmol/L) +DPI (10μmol/L) for 24 h. 1st—4th lane: CON,CON+DPI,CON+PD CON+DPI+PD; 5th—8th lane: HG,HG+DPI,HG+PD HG+DPI+PD. Observed band size: 85kDa.

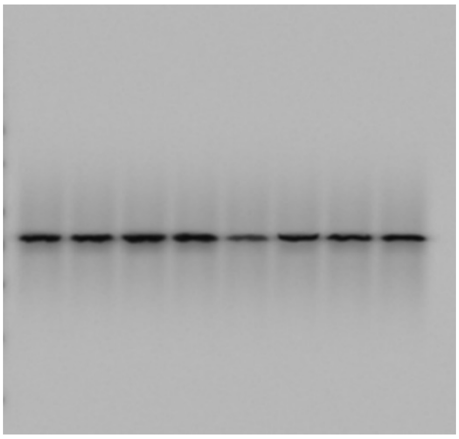

Figure 7G: GAPDH protein in the cytoplasm of H9c2 cells. H9c2 cells were cultured in DMEM containing 30 mmol/L glucose (HG) or 5.5 mmol/L glucose (normal glucose) and treated with polydatin (40 μmol/L), DPI (a NADPH oxidase inhibitor, 10μmol/L) or polydatin (40 μmol/L) +DPI (10μmol/L) for 24 h. 1st—4th lane: CON,CON+DPI,CON+PD CON+DPI+PD; 5th—8th lane: HG,HG+DPI,HG+PD HG+DPI+PD. Observed band size: 40kDa.

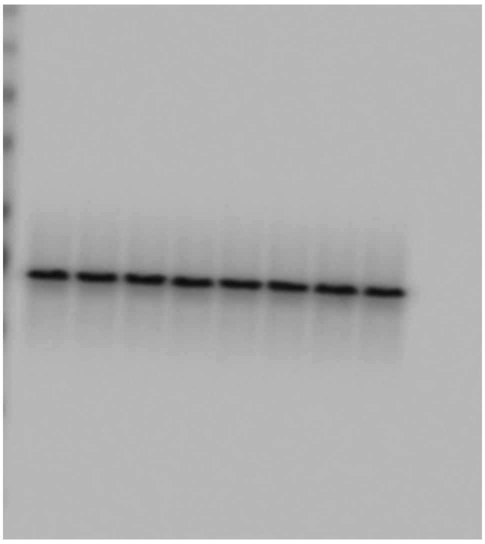

Figure 7G: p65 protein in nucleus of H9c2 cells. H9c2 cells were cultured in DMEM containing 30 mmol/L glucose (HG) or 5.5 mmol/L glucose (normal glucose) and treated with polydatin (40 μmol/L), DPI (a NADPH oxidase inhibitor, 10μmol/L) or polydatin (40 μmol/L) +DPI (10μmol/L) for 24 h. 1st—4th lane: CON,CON+DPI,CON+PD CON+DPI+PD; 5th—8th lane: HG,HG+DPI,HG+PD HG+DPI+PD. Observed band size: 65kDa.

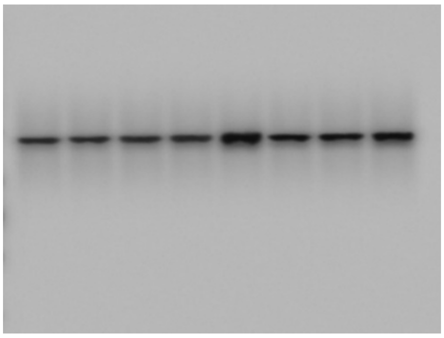

Figure 7G: Histone protein in nucleus of H9c2 cells. H9c2 cells were cultured in DMEM containing 30 mmol/L glucose (HG) or 5.5 mmol/L glucose (normal glucose) and treated with polydatin (40 µmol/L), DPI (a NADPH oxidase inhibitor, 10µmol/L) or polydatin (40 µmol/L) +DPI (10µmol/L) for 24 h. 1st—4th lane: CON,CON+DPI,CON+PD CON+DPI+PD; 5th—8th lane: HG,HG+DPI,HG+PD HG+DPI+PD. Observed band size: 15kDa.

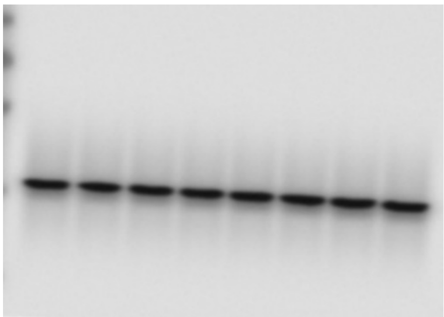

Supplement: Supplementary file 1 — Additional file 1. [file 12906_2020_3177_MOESM1_ESM.pdf]
